# Diabetic Neuropathy and Oxidative Stress: Therapeutic Perspectives

**DOI:** 10.1155/2013/168039

**Published:** 2013-04-24

**Authors:** Asieh Hosseini, Mohammad Abdollahi

**Affiliations:** ^1^Razi Drug Research Center, Iran University of Medical Sciences, Tehran 1449614535, Iran; ^2^Faculty of Pharmacy and Pharmaceutical Sciences Research Center, Tehran University of Medical Sciences, Tehran 1417614411, Iran

## Abstract

Diabetic neuropathy (DN) is a widespread disabling disorder comprising peripheral nerves' damage. DN develops on a background of hyperglycemia and an entangled metabolic imbalance, mainly oxidative stress. The majority of related pathways like polyol, advanced glycation end products, poly-ADP-ribose polymerase, hexosamine, and protein kinase c all originated from initial oxidative stress. To date, no absolute cure for DN has been defined; although some drugs are conventionally used, much more can be found if all pathophysiological links with oxidative stress would be taken into account. In this paper, although current therapies for DN have been reviewed, we have mainly focused on the links between DN and oxidative stress and therapies on the horizon, such as inhibitors of protein kinase C, aldose reductase, and advanced glycation. With reference to oxidative stress and the related pathways, the following new drugs are under study such as taurine, acetyl-L-carnitine, alpha lipoic acid, protein kinase C inhibitor (ruboxistaurin), aldose reductase inhibitors (fidarestat, epalrestat, ranirestat), advanced glycation end product inhibitors (benfotiamine, aspirin, aminoguanidine), the hexosamine pathway inhibitor (benfotiamine), inhibitor of poly ADP-ribose polymerase (nicotinamide), and angiotensin-converting enzyme inhibitor (trandolapril). The development of modern drugs to treat DN is a real challenge and needs intensive long-term comparative trials.

## 1. Introduction

A conduction problem arising in peripheral nerves is called peripheral neuropathy. Depending on the cause, the damage may appear in the axons or the myelin sheaths. The involved neurons may be afferent (sensory), efferent (motor), or both. The size of affected axons is an important issue, since sometimes only the small C unmyelinated and the A-delta fibers are affected. If these are damaged, symptoms move forward to pain sensors in the skin and autonomic neurons. Damage to large sensory fibers, which are the A-alpha and A-beta fibers, causes deficits in the proprioception and vibration sensation that results in muscle-stretch reflexes [1].

Diabetic neuropathy (DN), a microvascular complication of diabetes, comprises disorders of peripheral nerve in people with diabetes when other causes are ruled out. Diabetic peripheral neuropathy (DPN) is associated with considerable mortality, morbidity, and diminished quality of life [2]. The prevalence of neuropathy in diabetic patients is about 30%, whereas up to 50% of patients will certainly develop neuropathy during their disease [3]. In fact, against estimated universal prevalence of diabetes of 472 million by 2030, DPN is likely to affect 236 million persons worldwide causing lots of costs [4]. DPN can be broadly divided into generalized polyneuropathies and focal/multifocal varieties [5, 6]. The generalized form can be further classified into typical and atypical in terms of difference in onset, course, associations, clinical manifestations, and pathophysiology. The typical DPN is a chronic, symmetrical length-dependent sensorimotor polyneuropathy (DSPN) and the most common presentation of the peripheral nervous system damage by diabetes [7]. Therefore, considering the widespread of DN, it is vital to investigate details of its pathophysiology and therapeutic strategies. DN develops on a background of hyperglycemia and associated metabolic imbalances mainly oxidative stress. Hyperglycemia-induced overproduction of free radicals has been recognized as the source of further complications. Studies in the recent years have identified major pathways that are linked to DN, such as stimulated polyol, advanced formation of glycation end products, and other cascades of stress responses [8]. Since oxidative stress leads to such a major influence in the development of DN, in this paper we have highlighted the evidence linking DN, oxidative stress, and its consequences.

Despite efforts to make an early diagnosis and to stop the progression of DN, currently very few drugs are available to cure this disease and the others only provide symptomatic relief. Meanwhile, current goal of treatment of DN is to increase the functionality and quality of life and to diminish pain. In the present review, therapies on the horizon based on oxidative stress have been criticized.

## 2. Methods

Databases of PubMed, Google Scholar, Web of Science, Embase, Scopus, and DARE were searched up to 30 November 2012, for all relevant studies with DN. The search terms were diabetic neuropathy, oxidative stress, mechanisms, and current and new treatments without limiting search elements. All of relevant human (Table 1) and animal (Table 2) studies were included.

## 3. Clinical Features of DN

The most common form of DN is DSPN that accounts for a large proportion of all peripheral nerve manifestations attributed to diabetes, although, some physicians use the terms DSPN and DN interchangeably. Poor control of blood glucose is an important risk factor for the development of DN starting in the toes and gradually progressing proximally. Once it is diagnosed in the lower limbs, it may develop to the upper limbs with sensory loss [4]. For example, a patient with painful sensory neuropathy due to diabetes might first complain of burning or itchy sensations or even pain in the feet that is called paresthesias. The symptoms distribute on a so-called “glove and stocking” manner, as it starts from the longest axons. The unmyelinated nerve endings in the epidermis are degenerated first [2]. Neuropathic pain is the most disabling symptom observed in around one-third of patients with DN and about 20% of all diabetic patients. Painful DN deleteriously influences quality of life, sleep, mood, and the ability to work [4].

## 4. Pathogenesis of DN: Interaction of Oxidative Stress with Other Physiological Pathways

**Figure 1:**
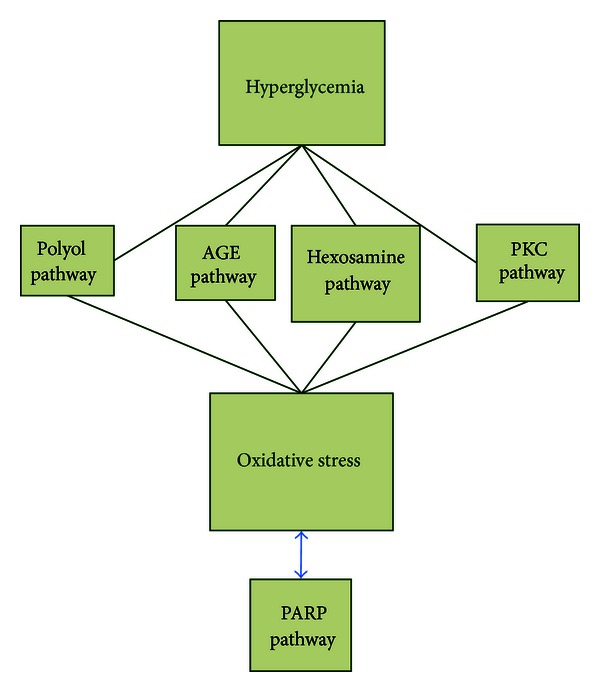
Interaction of oxidative stress with other physiological pathways in DN.

Although development of DN is multifactorial and the exact pathogenic mechanism is yet to be understood, a number of theories can be described. The current belief is that hyperglycemia, activation of polyol, advanced glycation end products (AGEs), hexosamine, diacylglycerol/protein kinase C (PKC), oxidative stress, nitric oxide, and inflammation all play key roles in DN. Based on evidence, oxidative stress is involved in all the above pathways (see Figure 1). These mechanisms are described one by one in the next sections of this paper, and then a conclusion is made.

### 4.1. Hyperglycemia

Excess intracellular glucose is processed by increased flux via one or more glucose metabolism pathways, and thus prolonged hyperglycemia results in progress of chronic complications of diabetes including DN.

### 4.2. Role of Polyol

**Figure 2:**
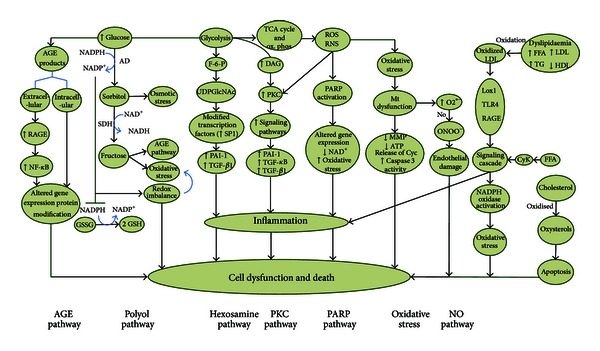
Mechanisms of diabetic neuropathy. The AGE and polyol pathways directly alter the redox capacity of the cell either through depletion of necessary components of glutathione recycling or by direct formation of ROS. The hexosamine, PKC, and PARP pathways indicate damage through expression of inflammation proteins. Dyslipidaemia with high incidence in T2D also linked to DN, and several underlying mechanisms have been identified. AGEs: advanced glycation end products; RAGEs: receptor for advanced glycation end products; NF-*κ*B: nuclear factor kappa B; AD: aldose reductase; SDH: sorbitol dehydrogenase; GSH: glutathione; GSSG: oxidized glutathione; F-6-P: fructose-6 phosphate; UDPGlcNAc: uridine diphosphate-N-acetylglucosamine; PAI-1: plasminogen activator inhibitor-1; TGF-*β*1: transforming growth factor-*β*1; DAG: diaceylglycerol; PKC: protein kinase C; ROS: reactive oxygen species; RNS: reactive nitrogen species; PARP: poly ADP-ribose polymerase; Mt: mitochondria; MMPs: mitochondrial membrane potentials; Cyc: cytochrome c; NO: nitric oxide; LDL: low-density lipoprotein; LOX1: oxidised LDL receptor 1; TLR4: toll-like receptor 4; FFA: free fatty acids; TG: triglycerides; HDL: high-density lipoprotein; CyK: cytokine.

Hyperglycemia results in elevated intracellular glucose in nerves, leading to saturation of the normal glycolytic pathway. Extra glucose lightens up the polyol pathway and that produces sorbitol and subsequently fructose by aldose reductase and sorbitol dehydrogenase, respectively. Increased polyol flux causes intracellular hyperosmolarity by an accumulation of impermeable sorbitol and compensatory efflux of other osmolytes such as myoinositol, taurine, and adenosine. In turn, shortage of myoinositol results in exhaustion of phosphatidylinositol and withdraws creation of adenosine triphosphate (ATP). All these processes result in a reduced activity of Na^+^/K^+^-ATPase and PKC, impaired axonal transport and structural breakdown of nerves, and finally presents itself as abnormal action potential. Aldose reductase-mediated reduction of glucose to sorbitol is associated with consumption of NADPH, and since NADPH is required for regeneration of reduced glutathione (GSH), this directly contributes to oxidative stress. In addition, formation of fructose from sorbitol promotes glycation, depletes NADPH, and increases AGEs which all result in major redox imbalance (see Figure 2) [9, 10].

### 4.3. Role of Advanced Glycation End Products (AGEs)

Hyperglycemia accelerates generation of AGEs via attachment of reactive carbohydrate groups to proteins, nucleic acids, or lipids. These groups tend to damage the biological task of proteins, which as a result affects cellular function. Extracellular AGEs also bind to the receptor of AGE (RAGE) and initiate inflammatory flows, activate NADPH oxidases, and generate oxidative stress. Long-term inflammatory responses upregulate RAGE and stimulate nuclear factor kappa B (NF*κ*B). Collectively, the biochemical damage induced by AGEs results in diminished neurotrophic support, impaired nerve blood flow, disrupted neuronal integrity, and impaired repair mechanisms (Figure 2) [3, 10].

### 4.4. Role of Hexosamine

The hexosamine, an additional factor, is implicated in the pathology of diabetes-induced oxidative stress and its complications. Fructose-6 phosphate, a metabolic intermediate of glycolysis, is shifted from the glycolytic pathway to the hexosamine way where fructose-6 phosphate is converted to uridine diphosphate-N-acetylglucosamine (UDPGlcNAc). Then, the UDPGlcNAc attaches to the serine and threonine residues of transcription factors. Hyperglycemic conditions create additional flux through hexosamine pathway that ultimately result in an increased activation of Sp1, a transcription factor implicated in diabetic complications. Activation of Sp1 leads to overexpression of transforming growth factor-*β*1 (TGF-*β*1) and plasminogen activator inhibitor-1 (PAI-1). The PAI-1 is upregulated by both hexosamine and PKC pathways. Collectively, activation of hexosamine pathway is implicated in multiple metabolic derangements in diabetes (see Figure 2) [9].

### 4.5. Activation of Diacylglycerol Protein Kinase C

Hyperglycemia stimulates formation of diacylglycerol, which then activates PKC. The PKC is an important element in function of nerves and pathogenesis of DN. Activation of PKC initiates an intracellular signaling cascade such as overexpression of PAI-1, NF-*κ*B, and TGF-*β*. It also increases the production of extracellular matrix and cytokines. Furthermore, it enhances contractility, permeability, and vascular endothelial cell proliferation such as motivation of cytosolic phospholipase A2 and inhibition of Na^+^/K^+^ ATPase (Figure 2) [9, 11]. PKC has a unique structural feature that according to redox status of cell facilitates its regulation. An antioxidant can react with catalytic domain to inhibit its activity, while the prooxidants react with regulatory domain to stimulate its activity. On activation, PKC triggers stress genes that phosphorylate transcription factors and thus alter the balance of gene expression resulting in oxidative stress [8].

### 4.6. Role of Oxidative Stress, Apoptosis, and Poly ADP-Ribose Polymerase

The generation of free radicals is a major factor in development of DN through increased glycolytic process. Oxidative stress and reactive oxygen species (ROS) link the physiological mediators and metabolic initiators implicated in progressive nerve fiber damage, dysfunction, and loss in DN. Simultaneous with generation of free radicals during the glycolytic process, oxidative stress harms mitochondrial DNA, proteins, and membranes [9, 12]. In fact, mitochondrial damage takes place due to surplus formation of ROS or reactive nitrogen species (RNS). Hyperglycemia induces mitochondrial changes such as release of cytochrome C, activation of caspase 3, altered biogenesis and fission, which all lead to a programmed cell death. Reduced mitochondrial action potentials (MMP) with modest ATP are resulted from thrilling entrance of glucose. This process results in surplus transport of oxidant electrons into the mitochondria. Neurotrophic support such as neurotrophin-3 (NT-3) and nerve growth factor (NGF) are also reduced by mitochondrial injury. It should be noted that axons are disposed to hyperglycemic hurt owing to their large content of mitochondria [12]. Oxidative stress in conjunction with hyperglycemia activates poly ADP-ribose polymerase (PARP) which further cleaves nicotinamide adenine dinucleotide (NAD^+^) to nicotinamide and ADP-ribose residues. This process continues by a link to nuclear proteins and results in changes of gene transcription and expression, NAD^+^ depletion, oxidative stress, and diversion of glycolytic intermediates to other pathogenic pathways such as PKC and AGEs (Figure 2) [9]. Collectively, the polyol, AGEs, PKC, hexosamine, and PARP, all contribute to neuronal damage together. The AGEs and polyol pathways openly modify the redox capacity of the cell either through weakening of necessary components of glutathione recycling or by direct construction of ROS. The hexosamine, PKC, and PARP pathways are representatives of damage mediated through expression of inflammatory proteins [9].

### 4.7. Nitric Oxide Deficiency/Impaired Endothelial Function

Vascular factors include impaired nerve perfusion, hypoxia, and nerve energetic defects that are all implicated in the pathogenesis of DN. Nerve blood flow is reduced in DN perhaps mediated via nitric oxide (NO). Overproduction of superoxide anion by the mitochondrial electron transport chain in DN leads to binding of this anion to NO to form the strong oxidant peroxynitrite which is right lethal to endothelial cells. Endothelial cells also produce NO that acts as a vasodilator and antagonizes thrombosis. The NO also defends against inflammation by adjusting (Na^+^/K^+^)-ATPase or inhibiting the production of potent vasoconstrictor peptide endothelin (ET)-1 [13, 14]. In addition, hyperhomocysteinemia is associated with impairment of endothelial function, providing a mechanism for its possible involvement in DN. There is a synergistic effect between AGEs and homocysteine in initiation of endothelial damage (Figure 2) [13].

### 4.8. Inflammation

The nerve tissues in diabetes undergo a proinflammatory process that presents symptoms and develops neuropathy. In addition, inflammatory agents such as C-reactive protein and tumor necrosis factor-*α* (TNF-*α*) are present in the blood of both type 1 diabetes (T1D) and type 2 diabetes (T2D) patients. The levels of C-reactive protein and TNF-*α* correlate with the incidence of neuropathy. Production of the initiating inflammatory mediators such as TNF-*α*, TGF-*β*, and NF-*κ*B results from several glucose-induced pathways. Cyclooxygenase-2 (COX-2) is an important enzyme that is upregulated by NF-*κ*B in diabetic peripheral nerves and consecutively generates prostaglandin E2 and ROS that trigger NF-*κ*B. Inducible nitric oxide synthase (iNOS) is an additional inflammatory enzyme which is regulated by NF-*κ*B. Similar to COX-2, iNOS either induces NF-*κ*B or is induced by it. This gives the impression that chronic NF-*κ*B activation is in the center of all the inflammatory elements operating in DN. Subsequent to ischemia reperfusion, an extensive and modest infiltration of macrophages and granulocytes takes place in diabetic peripheral nerves. The cytokines which are induced by NF-*κ*B in Schwann cells, endothelial cells, and neurons lead to absorption of macrophages in the diabetic nerves. Macrophages promote DN via a variety of mechanisms, including making of cytokines, ROS, and proteases, which all result in cellular oxidative damage and myelin breakdown. Excessive macrophage recruitment impairs regeneration of nerves in DN [9, 12].

### 4.9. Growth Factors

Neurotrophic factors play roles in the development, maintenance, and survival of neuronal tissue. In DN, the Schwann cells are damaged and neurons disintegrate, and the growth factors such as NGF, NT-3 and insulin-like growth factors (IGFs) are affected [9, 11].

## 5. Differences in the Pathophysiology of T1D and T2D in DN

As noted, hyperglycemia is a fundamental factor in DN. Dyslipidemia and changes in insulin signaling come after hyperglycemia in T2D. Levels of both insulin and C-peptide are reduced in patients with T1D while the neuronal insulin sensitivity is reduced in T2D. Therefore, the circumstances of disease in T1D and T2D are different, and this affects the efficacy of some medications [3].

### 5.1. Dyslipidemia

Dyslipidemia with high incidence in T2D is linked to DN. Free fatty acids have systemic effects such as release of inflammatory cytokine from adipocytes and macrophages. Plasma lipoproteins, especially low-density lipoproteins (LDLs), can be modified by glycation or oxidation where then binds to extracellular receptors comprising the oxidized LDL receptor 1(LOX1), toll-like receptor 4(TLR4), and RAGEs that activate NADPH oxidase. All these result in oxidative stress. Additionally, cholesterol can be simultaneously oxidized to oxysterols to cause neuronal apoptosis (Figure 2) [3].

### 5.2. Impaired Insulin Signaling

Insulin has neurotrophic effects and promotes neuronal growth and the survival while it is not involved in uptake of glucose into the neurons. Reduction of this neurotrophic signaling due to insulin resistance (T2D) or insulin deficiency (T1D) contributes to the pathogenesis of DN. In neurons, insulin resistance occurs by inhibition of the PI3K/Akt signalling pathway. Disruption of this pathway leads to mitochondrial dysfunction, oxidative stress, and dysfunction or death of the nerve. Tight glucose control is not as efficacious in patients with T2D, whereas it can reduce neuropathy in patients with T1D. This divergence is most likely related to differences in the underlying mechanisms in terms of hyperglycemia, dyslipidemia, and insulin resistance in T1D and T2D [3]. 

## 6. Treatment of DN

### 6.1. Control of Hyperglycemia

**Figure 3:**
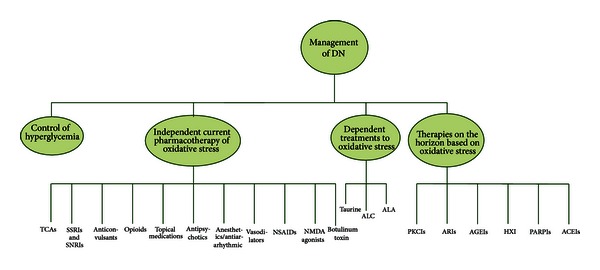
Algorithm for treatment of DN pain. TCAs: tricyclic antidepressants; SSRIs: selective serotonin reuptake inhibitors; SNRIs: serotonin norepinephrine reuptake inhibitors; NSAIDs: nonsteroidal anti-inflammatory drugs; ALC: acetyl-l-carnitine; ALA: *α*-lipoic acid; PKCIs: protein kinase C inhibitors; ARIs: aldose reductase inhibitors; AGEIs: advanced glycation end product inhibitors; GF: growth factor; PARPIs: poly ADP-ribose polymerase inhibitors; ACEIs: angiotensin converting enzyme inhibitors; HXIs: hexosamine pathway inhibitors.

As discussed above, hyperglycemia and/or insulin deficit and their concomitant actions are principally involved in the pathogenesis of DN. Thus, glycemic control gives the impression to be the most effective treatment to delay onset of DN and slowing its progress [14]. By contrast with the results obtained from patients with T1D, glucose control produces less definitive effect in patients with T2D. Therefore, despite the similarities between T1D and T2D, there are dissimilarities in the mechanisms and complications. It seems that glucose control is the only disease-modifying therapy for DN, as uncontrolled diabetes results in a pronounced oxidative stress that can be reversed if patients attain glycemic control. According to assumption that oxidative stress may mediate vascular, microvascular, and specific tissue complications in diabetes, antioxidant therapy remains a vital therapy [3]. Pain management is the other buttress of treatment for DN that can largely improve the quality of patients' life. As shown in Table 1, over the past two decades, enormous efforts have been made by doing randomized placebo-controlled trials to find effective treatments for DN. Based on these studies, several classes of drugs are considered to be effective for the treatment of DN, and also given the pathogenesis of DN, other drugs have been suggested as disease modifying all based on oxidative stress (Figure 3).

### 6.2. Current Pharmacotherapy of DN Independent of Oxidative Stress

#### 6.2.1. Tricyclic Antidepressants (TCAs)

The TCAs are recommended as the first-line therapy to relief pain of DPN for many years, even though they are not specifically endorsed for it. TCAs besides affecting catecholamines, they inhibit sodium and calcium channels, adenosine and N-methyl-D-aspartate (NMDA) receptors on the way to suppress neuronal hyperexcitability. TCAs have many side effects, principally anticholinergic effects [98, 99]. A meta-analysis based on number needed to treat (NNT) for TCAs resulted in the scores of 2 to 3 with a number needed to harm (NNH) of 14.7 [100].

#### 6.2.2. Selective Serotonin Reuptake Inhibitors (SSRIs) andSerotonin Norepinephrine Reuptake Inhibitors (SNRIs)

Because of the relative high rate of adverse effects and several contraindications of TCAs, the SSRIs can be considered for those who do not tolerate TCAs. The SSRIs inhibit presynaptic reuptake of serotonin but not norepinephrine, and unlike TCAs, they lack postsynaptic receptor blocking effects and quinidine-like membrane stabilization. There is limited evidence showing a beneficial role for SSRIs, as they have not been licensed for the treatment of DN pain [14, 99, 101]. Antidepressants with dual selective inhibition of serotonin and norepinephrine (SNRIs) such as duloxetine and venlafaxine are better. SNRIs block the noradrenaline and 5-HT transporters and inhibit monoamine reuptake from the synaptic cleft into the presynaptic terminal which finally result in inhibition of excitatory impulse and pain perception [98, 101–103].

#### 6.2.3. Anticonvulsants

Gabapentin and pregabalin should be used as first-line treatment for DPN pain if there is inadequate response or contraindications to TCAs. Pregabalin and gabapentin bind to the alpha2-delta subunit of the calcium-sensitive channels on the presynaptic neuron and modulate neurotransmitter release [99, 100]. Based on the new guidelines, pregabalin is recommended for the treatment of painful DN [102, 103]. Sodium valproate is probably effective in lessening pain and should be considered for the treatment of painful DN, but its adverse effects are high. For instance, it can worsen glycemic control and weight gain. Lamotrigine, oxcarbazepine and lacosamide should probably not be considered for the treatment of painful DN. Topiramate also lacks adequate support to be used in DN [103].

#### 6.2.4. Opioids

Opioids by interacting with receptors located on neuronal cell membranes prevent neurotransmitter release at the presynaptic nerve terminal and reduce pain [14]. Controlled-release oxycodone, tramadol, and morphine can be exampled [99]. A meta-analysis indicated an NNT of 2.6 for oxycodone, 3.9 for tramadol, and 2.5 for morphine [100] in the treatment of painful DN [103].

#### 6.2.5. Topical Medications

Current topical treatments for DPN pain include capsaicin cream and lidocaine 5% patches. This combination stimulates C fibers to release, and subsequently deplete substance P. Capsaicin application causes degeneration of epidermal nerve fibers as a mechanism of its analgesic effect, and thus caution for its use in insensitive diabetic foot is needed [14]. In a 2009 pooled data analysis, the calculated NNT for capsaicin was 5.7 [53]. Lidocaine blocks voltage-gated channels in damaged nerves [99]. There have been small effective trials with lidocaine. A randomized controlled trial (RCT) in 2003 revealed an NNT of 4.4 for it [67]. Topical clonidine is probably not effective and should not be considered [103].

#### 6.2.6. Antipsychotics

Few atypical antipsychotics have been introduced for painful DN. The newer, less dopamine selective antipsychotics was supported by some animal studies consistent with clinical trials [104]. These compounds may induce negative metabolic effects such as weight gain and insulin resistance [105].

#### 6.2.7. Anesthetics/Antiarrhythmic

These agents are all potent voltage-gated sodium channel antagonists and perhaps are less used in DN [105]. Mexiletine, lidocaine, and tocainide have all shown benefits for painful DN in smaller RCTs. Although tocainide has positive effects, but caution is needed for its cardiotoxicity [105]. Mexiletine has not been found effective [103]. The easiest method to incorporate the utility of the anesthetic/antiarrhythmics in patients with painful DN is the use of 5% lidocaine patches [105].

#### 6.2.8. Vasodilators

Isosorbide dinitrate nasal spray and glyceryl trinitrate transdermal patches have shown positive effects in painful DN [105].

#### 6.2.9. Nonsteroidal Anti-Inflammatory Drugs (NSAIDs)

In patients with acute painful peripheral neuropathy, simple analgesics such as NSAIDs may provide pain control by modulating the nociceptive and inflammatory pain pathways [14]. 

#### 6.2.10. NMDA Antagonists

Dextromethorphan, a low affinity NMDA receptor blocker, has been effective in relieving pain and improving quality of life in patients with DN [103].

#### 6.2.11. Botulinum Toxin

This toxin not only works on the acetylcholine channels but also involves other mechanisms. Existing studies lack large number of subjects, and more is required to overcome doubt and debate about efficacy of this toxin [105].

### 6.3. Treatments Dependent on Oxidative Stress

The strongest sign for the role of oxidative stress in DN is the studies that report positive effects of antioxidants in both animal models and patients. Although, it is impossible to review all the antioxidants that can be effective to prevent or delay the onset of DN, some can be listed such as acetyl-L-carnitine, taurine, *β*-carotene, free amino acids, vitamin E, curcumin, ascorbic acid, and lipoic acid [8].

#### 6.3.1. Taurine

Taurine is an antioxidant with effects on neuronal calcium signaling. Taurine improves electrophysiological parameters and nerve blood flow and exhibits analgesic properties in patients with DN [14].

#### 6.3.2. Acetyl-L-Carnitine

Acetyl-L-carnitine (ALC), the acetylated ester of the amino acid L-carnitine, as an antioxidant has shown significant reduction in pain of patients with DN [14].

#### 6.3.3. Alpha Lipoic Acid

D-L-*α*-lipoic acid (ALA) is a potent antioxidant, which has been extensively evaluated in subjects with DN and has shown good effects [14]. But based on new guidelines, there is insufficient evidence to recommend it in treatment of DN [103].

### 6.4. Nonpharmacologic Agents

Percutaneous electrical nerve stimulation is probably effective in reducing pain and improving sleep in patients who have painful DN. Exercise and acupuncture lack evidence of efficacy in the treatment of painful DN. Low-intensity laser therapy, Reiki therapy, and electromagnetic field treatment (such as magnetized shoe insoles) are probably not effective and should not be considered [103].

### 6.5. Therapies on the Horizon Based on Oxidative Stress

Since constant tight glucose control is difficult and still a challenge in most cases, additional therapies that target the hyperglycemia-induced complications are now under attention of researchers. To this end, coping against some known pathways that are activated as a consequence of increased oxidative stress and glucose flux is deemed effective to control DN. These pathways and inhibition of them are reviewed below.

#### 6.5.1. Protein Kinase C Inhibitors

It is thought that activation of PKC through hyperglycemia-induced oxidative stress contributes to the generation of free radicals that result in diabetic microvascular complications [14]. Some of the PKC inhibitors such as ruboxistaurin have been shown to exhibit antioxidant effects. Ruboxistaurin has been particularly successful in reducing the progress of DN, and it is pending to get official approval [106].

#### 6.5.2. Aldose Reductase Inhibitors (ARIs)

As stated earlier, hyperglycemia-mediated activation of the polyol pathway can produce oxidative stress that may underlie DN. Aldose reductase is a key enzyme in pathogenesis of DN. ARIs drop the flux of glucose through the polyol or sorbitol pathways resulting in a reduction of intracellular accumulation of sorbitol and fructose [14]. The effects of fidarestat (a novel ARIs) on nerve conduction and the subjective symptoms of DN provided evidence that this treatment can control DN with concomitant reduction in oxidative stress markers [107]. Similarly long-term treatment with epalrestat, an ARI, can effectively delay the progress of DN and improve the symptoms, particularly in subjects with limited microangiopathy and good glycemic control [108]. Treatment with ranirestat (ARIs) also appears to have an effect on motor nerve function in mild to moderate diabetic sensorimotor polyneuropathy (DSP), but the results of this study did not show a statistically significant difference in sensory nerve function relative to placebo [109].

#### 6.5.3. Advanced Glycation End Product Inhibitors (AGEIs)

Accumulation of AGEs and activation of AGE receptors lead to oxidative stress and microvascular damage in DN. Benfotiamine, a derivative of thiamine (vitamin B1), reduces tissue AGEs formation and oxidative stress in subjects with DN with varying doses and duration of treatment [14, 110]. Also aspirin, because of its antioxidant capacity, and aminoguanidine, a free radical scavenger, can inhibit this pathway and reduce oxidative stress in DN [9].

#### 6.5.4. Hexosamine Pathway Inhibitors

As described above, diabetes-induced oxidative stress stimulates hexosamine pathway that implicates in the pathology of DN. Benfotiamine decreases flux through hexosamine pathway resulting in lower oxidative stress. This agent can reduce the pain associated with DN [9].

#### 6.5.5. Inhibition of PARP

As discussed above, PARP activation involves in the pathogenesis of DN, and its inhibition lightens numerous experimental pathologic conditions connected with oxidative stress in DN. Nicotinamide has been shown to act as a PARP inhibitor and an antioxidant in animals that improves complications of early DN [9].

#### 6.5.6. Angiotensin Converting Enzyme Inhibitors

Angiotensin II is a potent vasoconstrictor with proinflammatory properties, which especially in the absence of NO causes thrombosis. It also stimulates intercellular adhesion molecules and vascular adhesion molecules (VCAMs). The role of angiotensin converting enzyme inhibitors in DN is probably through inhibition of angiotensin II. In this regard, trandolapril has shown a small but significant improvement in some complication of DN [14].

## 7. New Therapeutic Approaches for DN

Despite relative lack of success of interventional agents to reverse or slow established DN, there is still hope to find some good agents. We have summarized a few of new approaches in Table 2 [79–97]. Hopefully, some reviews in the recent years have proven positive effects of natural antioxidants and herbal products such as *Satureja* species and *Urtica dioica* in diabetes and its complications [111–116].

## 8. Conclusion

In the present review, we tried to elaborate the pathogenesis of disease with a focus on oxidative stress and introduced therapies dependent or independent of oxidative stress. Diabetes can injure peripheral nerves in various distributions, and DSPN is the most common presentation in diabetes, which lead to substantial pain, morbidity, and impaired quality of life. Social and health-care costs linked with DN are high. DN develops on a background of hyperglycemia and associated metabolic imbalance. Numerous biochemical mechanisms of neurovascular and nerve damage have been identified in DN, but excessive production of ROS or oxidative stress is thought to be a common etiologic factor.

Treatment of DN always begins with optimizing glycemic control and then control of pain. Regarding role of oxidative stress and consequential factors in pathogenesis of DN, observing positive results with inhibitors of key pathways at the preclinical level is not surprising, but final decision will be made on the basis of clinical trials. If oxidative stress is assumed only as an ancillary player in DN, then antioxidants should be supplemented with conventional treatments. Development of new drugs to treat DN still remains a challenge that needs intensive long-term comparative trials.

## Figures and Tables

**Table 1:** Current pharmacotherapy in DN.

NNT	Study outcome	Treatment duration	Study design	Daily dose (mg)	Trial size	Trial	Drug used	Drug class
—	Amitriptyline > placebo	2 × 6 wk	Crossover	Up to 150 mg	29	Max [15]	Amitriptyline	Antidepressants: TCAs:
2.1	Amitriptyline > placebo	2 × 6 wk	Crossover	≤150 mg	29	Max et al. [16]	Amitriptyline	
2.2	Amitriptyline = desipramine > placebo	2 × 6 wk	Crossover	Amitriptyline: 105 mg; desipramine: 111 mg	38	Max et al. [17]	Amitriptyline and desipramine	
—	Amitriptyline > maprotiline > placebo	4 wk	Crossover	75 mg	37	Vrethem et al. [18]	Amitriptyline and maprotiline	
—	Amitriptyline > placebo	2 × 6 wk	Crossover	25–75 mg	24	Morello et al. [19]	Amitriptyline	
—	Clomipramine > desipramine > placebo	6 wk	Crossover	Desipramine: 200 mg and clomipramine: 75 mg (in extensive metabolisers). 50 mg of both drugs (in poor metabolisers)	19	Sindrup et al. [20]	Desipramine and clomipramine	
	Desipramine > placebo	6 wk	Crossover	201 mg	20	Max et al. [21]	Desipramine	
—	Imipramine > placebo	5 + 5 wk	Crossover	100 mg	12	Kvinesdal et al. [22]	Imipramine	
—	Combination > placebo	8 wk	Crossover	Nortriptyline: 10 mg; fluphenazine: 0.5 mg	18	Gomez-Perez et al. [23]	Nortriptyline and fluphenazine	

—	Citalopram > placebo	2 × 3 wk	Crossover	40 mg	15	Sindrup et al. [24]	Citalopram	SSRI:

—	Venlafaxine + gabapentin > placebo in patients who do not respond to gabapentin	2 × 8 wk	Parallel	—	11 and 42	Simpson [25]	Venlafaxine and gabapentin	SNRIs
5.2 for venlafaxine and 2.7 for imipramine.	Venlafaxine > imipramine > placebo	4 wk	Crossover	Venlafaxine: 225 mg; imipramine: 150 mg	29	Sindrup et al. [26]	Venlafaxine versus imipramine	

4.5	Venlafaxine > placebo	6 wk	Parallel	150–225 mg	244	Rowbotham et al. [27]	Venlafaxine	
—	Venlafaxine > placebo	8 wk	Parallel	75–150 mg	60	Kadiroglu et al. [28]	Venlafaxine	
11 (60 mg group); 5 (120 mg group)	Duloxetine > placebo	12 wk	Parallel	60, 120 mg	348	Raskin et al. [29]	Duloxetine	
4.3 (60 mg group); 3.8 (120 mg group)	Duloxetine > placebo	12 wk	Parallel	20, 60, 120 mg	457	Goldstein et al. [30]	Duloxetine	
6.3 (60 mg group); 3.8 (120 mg group)	Duloxetine > placebo	12 wk	Parallel	60, 120 mg	334	Wernicke et al. [31]	Duloxetine	
5.2 and 4.9 (duloxetine 60 mg once daily and 60 mg BID, resp.)	Duloxetine > placebo	3 × 12 wk	Parallel	60 mg	1024	Kajdasz et al. [32]	Duloxetine	
3.6 (300 mg group); 3.3 (600 mg group)	Pregabalin (300, 600 mg) > placebo	5 wk	Parallel	75, 300, 600 mg	338	Lesser et al. [33]	Pregabalin	
3.9	Pregabalin > placebo	8 wk	Parallel	300 mg	146	Rosenstock et al. [34]	Pregabalin	
4.2 (600 mg group)	Pregabalin (600 mg) > placebo	6 wk	Parallel	150, 600 mg	246	Richter et al. [35]	Pregabalin	
3.6	Flexible and fixed > placebo	12 wk	Parallel	Flexible: 150, 300, 450, 600 mg; fixed: 300, 600 mg	338	Freynhagen et al. [36]	Pregabalin	
6.3 (600 mg group)	Pregabalin (600 mg) > placebo	12 wk	Parallel	150, 300, or 600 mg	395	Tölle et al. [37]	Pregabalin	
—	Pregabalin > placebo	13 wk	Parallel	600 mg	167	Arezzo et al. [38]	Pregabalin	
4.04 (600 mg group); 5.99 (300 mg group); 19.06 (150 mg group)	150, 300, 600 mg TID > placebo; 600 mg BID > placebo	5 to 13 wk	Parallel	150, 300, 600 mg administered TID or BID	—	Freeman et al. [39]	Pregabalin	
4	Gabapentin > placebo	8 wk	Parallel	Titrated from 900 to 3600 mg	165	Backonja et al. [40]	Gabapentin	
—	Gabapentin = placebo	2 × 6 wk	Crossover	900 mg	40	Gorson et al. [41]	Gabapentin	
—	Sodium valproate > placebo	4 wk	Parallel	600–1200 mg	52	Kochar et al. [42]	Sodium valproate	
—	Sodium valproate > placebo	16 wk	Parallel	500 mg	39	Kochar et al. [43]	Sodium valproate	Anticonvulsants:
—	Sodium valproate = placebo	4 wk	Crossover	1500 mg	31	Otto et al. [44]	Sodium valproate	
4	Lamotrigine > placebo	6 wk	Parallel	Titrated from 25 to 400 mg	59	Eisenberg et al. [45]	Lamotrigine	
—	Lamotrigine = placebo	19 wk	Parallel	200, 300, 400 mg	360	Vinik et al. [46]	Lamotrigine	
—	Lamotrigine = amitriptyline	6 wk	Crossover,	Lamotrigine: 25, 50, 100 mg twice daily; amitriptyline: 10, 25, 50 mg at night time	53	Jose et al. [47]	Lamotrigine and amitriptyline	
—	Carbamazepine > placebo	2 wk	Crossover	200–600 mg	30	Rull et al. [48]	Carbamazepine	
—	Oxcarbazepine > placebo	16 wk	Parallel	300 mg titrated to a maximum dose of 1800 mg	146	Dogra et al. 2005 [49]	Oxcarbazepine	
7.9 (1200 groups); 8.3 (1800 groups)	Oxcarbazepine > placebo (1200, 1800 mg groups)	16 wk	Parallel	600, 1200, 1800 mg	347	Beydoun et al. [50]	Oxcarbazepine	
—	Oxcarbazepine = placebo	16 wk	Parallel	1200 mg	141	Grosskopf et al. [51]	Oxcarbazepine	
—	Lacosamide > placebo	—	Parallel	400 mg	94	Rauck et al. [52]	Lacosamide	
—	Lacosamide (400 mg group) > placebo	18 wk	Parallel	200, 400, 600 mg	—	Wymer et al. [53]	Lacosamide	

3.1	Tramadol > placebo	6 wk	Parallel	210 mg	131	Harati et al. [54]	Tramadol	
4.3	Tramadol > placebo	2 × 4 wk	Crossover	200–400 mg	45	Sindrup et al. [55]	Tramadol	
—	Tramadol/acetaminophen > placebo	8 wk	Parallel	Tramadol: 37.5 mg; acetaminophen: 325 mg	311	Freeman et al. [56]	Tramadol/ acetaminophen	
—	Oxycodone > placebo	6 wk	Parallel	10–100 mg	159	Gimbel et al. [57]	Oxycodone	Opioids:
2.6	Oxycodone > placebo	4 wk	Crossover	10–80 mg	45	Watson et al. [58]	Oxycodone	
—	Oxycodone + gabapentin > placebo + gabapentin	12 wk	Parallel	Oxycodone: 10–80 mg + gabapentin: 100–3600 mg	338	Hanna et al. [59]	Oxycodone	
—	Morphine + gabapentin > morphine > gabapentin > placebo	4 × 4 wk	Crossover	120, 60 mg morphine + 2400 mg gabapentin, 3600 mg gabapentin	57	Gilron et al. [60]	Morphine	
—	Capsaicin > vehicle	8 wk	Parallel	0.075% capsaicin	252	Anonymous et al. [61]	Capsaicin	
—	Capsaicin > vehicle	8 wk	Parallel	0.075% capsaicin	—	Scheffler et al. [62]	Capsaicin	
—	Capsaicin > vehicle	8 wk	Parallel	0.075% capsaicin four times a day	22	Tandan et al. [63]	Capsaicin	
—	Capsaicin > vehicle	8 wk	Parallel	0.075% capsaicin four times a day		Anonymous et al. [64]	Capsaicin	Topical medications:
—	Isosorbide > placebo	2 × 4 wk	Crossover	30 mg	22	Yuen et al. [65]	Isosorbide dinitrate spray	
—	Glyceryl > placebo	2 × 4 wk	Crossover	—	48	Agrawal et al. [66]	Glyceryl trinitrate spray	
4.4	Lidocaine > placebo	4 wk	Crossover	5% lidocaine patch	40	Meier et al. [67]	Lidocaine patch	
—	Lidocaine significantly improved pain and quality of life	3 wk study with a 5 wk extension	Open label, flexible dosing	5% lidocaine patch	56	Barbano et al. [68]	Lidocaine patch	

—	Mexiletine > placebo	10 wk	Crossover	10 mg	16	Dejgard et al. [69]	Mexiletine	Anesthetics/ antiarrhythmics:
	Mexiletine > placebo	3 wk	Parallel	675 mg	216	Oskarsson et al. [70]	Mexiletine	
—	Mexiletine = placebo	3 wk	Parallel	600 mg	29	Wright et al. [71]	Mexiletine	

4	Dextromethorphan > placebo	2 × 6 wk	Crossover	Mean 381 mg	14	Nelson et al. [72]	Dextromethorphan	NMDA antagonists:
3.2	Dextromethorphan > placebo	2 × 9 wk	Crossover	400 mg	19	Sang et al. [73]	Dextromethorphan	

3.03 at 12 weeks	Botulinum toxin > placebo	24 wk	Parallel	Intradermal of subtype A (20–190 units) into the painful area	29	Ranoux et al. [74]	Botulinum toxin	Other drugs:
—	Botulinum toxin > placebo	12 × 12 wk	Crossover	50 units of subtype A in 1.2 mL 0.9% saline given intradermally into each foot, each injection 4 U subtype A	18	Yuan et al. [75]	Botulinum toxin	
—	Improved pain and nerve fiber regeneration	2 × 52 wk	Parallel	500 and 1,000 mg, three times per day	—	Sima et al. [76]	Acetyl-L-carnitine	
—	*α*-lipoic acid = placebo	28 wk	Parallel	600 mg	509	Ziegler et al. [77]	*α*-lipoic acid	
—	*α*-lipoic acid ≥ placebo with clinically meaningful degree	3 wk	Parallel	600 mg	1258	Ziegler et al. [78]	*α*-lipoic acid	

NNT: number needed to treat; TCAs: tricyclic antidepressants; SSRI: selective serotonin reuptake inhibitor; SNRIs: serotonin norepinephrine reuptake inhibitors; NMDA: N-methyl-D-aspartate; TID: three times daily; BID: twice daily.

**Table 2:** New therapeutic approaches for DN. DRG: dorsal root ganglion neuron.

Endpoint	Study populations	Compound	Study
Improvement of peripheral nerve function	Diabetic rats	Salvianolic acid A	Yu et al. [79]
Improvement of DN	Animal model of T2D	High-fat diet with menhaden oil	Coppey et al. [80]
Improvement of DN	Patients with T2D and neuropathy	Tai Chi exercise	Ahn and Song [81]
Improvement of DN	T2DM patients	Beraprost sodium	Shin et al. [82]
Improvement of DN	STZ-diabetic rats	Anandamide	Schreiber et al. [83]
Improvement of peripheral nerve function	Mouse model of DPN	Thymosin *β*4	Wang et al. [84]
Improvement of chronic pain, including PDN	Rat model of STZ-induced PDN	Gastrodin	Sun et al. [85]
Prevention of progression of DN	Patients enrolled in the aldose reductase inhibitor-diabetes complications	Epalrestat	Hotta et al. [86]
Improvement of DN	STZ-diabetic rats	Gliclazide with curcumin	Attia et al. [87]
Improvement of DN	STZ-diabetic rats	Bone marrow-derived mononuclear cells	Naruse et al. [88]
Neuroprotection effect	In vitro model of high glucose-treated DRG neurons in culture	Galanin	Xu et al. [89]
Improvement of DN	—	Baicalein	Yorek [90]
Improvement of neuropathic pain	Animal models of neuropathic pain	Brazilian armed spider venom toxin Tx3-3	Dalmolin et al., [91]
Neuroprotection effect Improvement of DN	STZ-diabetic rats	Magnesium-25 carrying porphyrin-fullerene nanoparticles	Hosseini et al. [92, 93]
Maintaining health in diabetes	STZ-diabetic rats	Phosphodiesterase inhibitors	Milani et al. [94]
Improve transplant outcome and graft function in diabetes	Isolated rat pancreatic islets	IMOD	Larijani et al. [95]
Improve islet transplantation in diabetes	Isolated rat pancreatic islets	Cerium and yttrium oxide nanoparticles	Hosseini and Abdollahi [96, 97]
